#  Evaluation of vacuum phenomenon in dogs with coxofemoral degenerative joint disease using computed tomography

**DOI:** 10.1186/s12917-020-02485-2

**Published:** 2020-08-06

**Authors:** Kanokporn Kanthavichit, Auraiwan Klaengkaew, Naparee Srisowanna, Nardtiwa Chaivoravitsakul, Kongthit Horoongruang, Chutimon Thanaboonnipat, Kumpanart Soontornvipart, Nan Choisunirachon

**Affiliations:** 1 Department of Veterinary Surgery, Faculty of Veterinary Science, Chulalongkorn University, 10330 Bangkok, Thailand; 2 The Small Animal Teaching Hospital, Faculty of Veterinary Science, Chulalongkorn University, 10330 Bangkok, Thailand

**Keywords:** computed tomography, degenerative joint disease, dog, hip, vacuum phenomenon

## Abstract

**Background:**

Degenerative joint disease (DJD) is a common orthopedic lesion in the coxofemoral joint of canine patients. Concurrent with the sign of degeneration, the vacuum phenomenon (VP), or intra-articular gas, has been observed in several locations in both human and canine patients. A cadaveric study described VP in small breed dogs without DJD but with hip laxity. However, none of the canine VP reports mentioned coxofemoral DJD. Therefore, the aim of this retrospective study was to describe the distribution of coxofemoral VP revealed on computed tomography (CT) imaging in canine patients with DJD.

**Results:**

A total of 282 dogs (564 coxofemoral joints), comprising 142 small, 85 large, and 55 medium breeds, were included in the present study. DJD was found in 31.02% of all dogs. The incidence of DJD was highest in large breed dogs (50%), followed by medium (31.81%) and small (19.36%) breed dogs. In addition, VP was detected with CT using the pulmonary window in 31 joints of 25 dogs that received a diagnosis of hip degenerative disease. VP was found most frequently at the laterodorsal area of the acetabulum. The incidences of VP in large, small, and medium breed dogs were 35.94%, 17.14% and 8.33%, respectively. The presence of coxofemoral VP was significantly and positively correlated with DJD (odds ratio = 17.58, 95% CI 2.32–133.42).

**Conclusions:**

The presence of coxofemoral joint DJD and VP was more likely to be detected in large breed dogs, especially in those with established DJD. CT is an advanced diagnostic imaging modality that can be used to reveal VP lesions, most of which are reported at the laterodorsal acetabulum. Further studies, including comparisons of different patient positions, may reveal more information regarding coxofemoral VP.

## Background

Degenerative joint disease (DJD) is the most common orthopedic disease reported in dogs, especially in large to giant breeds [1]. The cause of DJD is unknown, but several factors, including hip laxity, obesity and genetic factors, have been hypothesized [2–5]. Moreover, angular limb deformity, septic arthritis, and cranial cruciate ligament rupture have been reported [6]. Radiography is generally performed to make a definitive diagnosis of DJD, ranging from early- to severe-stage DJD, on the basis of the lesions detected [7]. Currently, more advanced imaging modalities are available for detecting articular degenerative changes, including ultrasonography, computed tomography (CT), and magnetic resonance imaging (MRI) [7–9]. Some studies have reported that two- and three-dimensional CT imaging can assist veterinarians in predicting joint osteoarthritis because these modalities can reveal volumetric changes in articular components such as the acetabulum and femoral head [7]. These advanced diagnostic modalities are currently being used to obtain additional information regarding DJD, especially in dogs [7].

In canine patients, DJD frequently affects the coxofemoral joint [10]. Studies have reported that age, overweight, and the presence of canine hip dysplasia (CHD) may be risk factors for coxofemoral DJD [3, 11]. In addition to bony changes, gas in the intra-articular space of the DJD joint can be detected on a radiograph or CT imaging scan [8, 12, 13]. The presence of intra-articular gas was first described in a human patient by Fick in 1910 [14], and it was later termed the “vacuum phenomenon” (VP) [15]. This phenomenon is characterized by an accumulation of gas in a synovial joint of the appendicular skeleton or an area of the spine. Synovial gas is mostly related to DJD in humans, but it can be found in other pathologies, such as bone fracture, metastasis and abscess [16]. However, the stress position and traction may lead to VP, as reported in sound children [17]. In synovial joints (arthrodial) under traction, it is assumed that intra-articular gas, comprised mainly of nitrogen, oxygen, carbon, and other gases, originates from tissues surrounding the joint, and these gases are unable to dissolve in the joint space and synovial fluid [18]. VP in humans is commonly observed at several locations, such as the temporomandibular joints [13], knee [19], sacroiliac joints [20] and coxofemoral joints, which are affected by femoroacetabular impingement (FAI) [21] or developmental dysplasia of the hip (DDH) [17]. In contrast, in animals, VP was detected at metatarsophalangeal joints [22], scapulohumeral joints [23], and lumbosacral joints [24]. Furthermore, VP can be observed on X-ray examination of the coxofemoral joint in an extended position or PennHIP distraction [5, 25]. Although DJD is most commonly reported to affect the coxofemoral joint in canine patients [26], the presence of VP in the coxofemoral joint DJD has not been reported. Therefore, the aim of this study was to determine the prevalence, characteristics, and relationship between VP and coxofemoral DJD in dogs using CT imaging. Our hypothesis was that VP can be diagnosed in dogs with coxofemoral joint DJD.

## Results

### Clinical demographic data

Between May 2013 and April 2018, 1,200 canine patients underwent CT; however, only 282 met the inclusion criteria for this study. These 282 dogs included a total of 564 coxofemoral joints and 33 canine breeds. The dogs were classified into three groups based on the American Kennel Club (AKC): small (142/282 dogs or 284/564 joints; 50.35%), medium (55/282 dogs or 110/564 joints; 19.50%), and large (85/282 dogs or 170/564 joints; 30.14%) breeds. The most common breeds in this study were Chihuahua (33/282; 11.70%), Shih Tzu (32/282; 11.34%), Pomeranian (28/282; 9.92%), golden retriever (19/282; 6.74%), and poodle (18/282; 6.38%). The median age of the dogs was 7 years (range, 6 months to 17 years). There were 158 male (116 intact, 42 castrated) and 124 female (71 intact, 53 spayed) dogs, with a mean body weight of 15.04 ± 12.49 kg (range, 1.00–60.00 kg; median, 10 kg). The gonadal status, body weight, and median age of each canine breed group are included in Table 1.

**Table 1:** Clinical data and prevalence of degenerative joint disease of hip joint and vacuum phenomenon in dogs

	Total hip	Median age(range)	Body Weight (mean ±SD)	DJD score(hip)			Vacuum phenomenon (hip)			Odds ratio (CI)	*P-*value
				Grade 0	Grade 1	Grade 2	Normal	Grade1	Grade2		
All(n=282)	564	7(0.5 –17)	15.04 ±12.49	389	52	123	0	0	31	11.89 (1.61-87.87)	0.015*
Small breeds(n=142)	284	6(0.5 -17)	5.14 ±2.89	229	20	35	0	0	6	1.5 (0.18 -12.65)	0.697
Medium breeds(n=55)	110	10(0.5 -16)	13.69 ±4.17	75	11	24	0	0	2	0.95 (0.09-9.54)	0.963
Large breeds(n=85)	170	8(0.5 -16)	30.89 ±8.21	85	21	64	0	0	23	17.58 (2.32-133.42)	0.005*

*P* probability, *CI* confidence interval, * = significant (*P* <0.05), *NS* not significant (*P* > 0.05)

Grade 0 = normal, Grade 1 = mild, Grade 2 = moderate to severe

### CT imaging and coxofemoral DJD

Among all dogs, 219 dogs underwent CT scanning in the prone position, whereas 60 and 3 underwent CT in the supine and lateral positions, respectively. Among the 564 joints of the 282 included dogs, 175 (31.02%) were diagnosed with DJD of the coxofemoral joint. According to DJD grading, 389 (68.97%) joints were scored as G0 (no lesion), 52 (9.21%) as G1 (developing coxofemoral DJD), and 123 (21.80%) as G2 (established coxofemoral DJD). The presence of coxofemoral DJD was more frequently detected in large breed dogs (85 of 170 joints had DJD [50.00%]; G1 = 21 joints, G2 = 64 joints) followed by medium (35 of 110 joints had DJD [31.81%]; G1 = 11 joints, G2 = 24 joints) and small (55 of 284 joints had DJD [19.36%]; G1 = 20 joint, G2 = 35 joints) breed dogs. Information regarding DJD scores according to the breed of the canine patients is summarized in Table 1. A significant association between age and the presence of coxofemoral DJD was detected in all dogs, large breed dogs and small breed dogs (*P* = 0.0018, *P* = 0.0127, and *P* = 0.0086, respectively), but not in medium breed dogs (*P* = 0.1035).

### Evidence of coxofemoral VP on CT imaging

VP was detected only in dogs affected by DJD: 31/175 (17.71%) joints of 25 examined patients. It was seen only in G2 DJD, which accounted for 31/123 (25.20%). Comparing clinical and demographic information, VP was detected in dogs ranging in age from 1 to 15 years (median, 11 years). No association between age and the presence of coxofemoral VP was detected in any of the enrolled dogs (P = 0.5645). The sex distribution of VP was equal within the sample: 13 males (9 intact, 4 castrated) and 12 females (9 intact, 3 spayed).

**Fig. 1:**
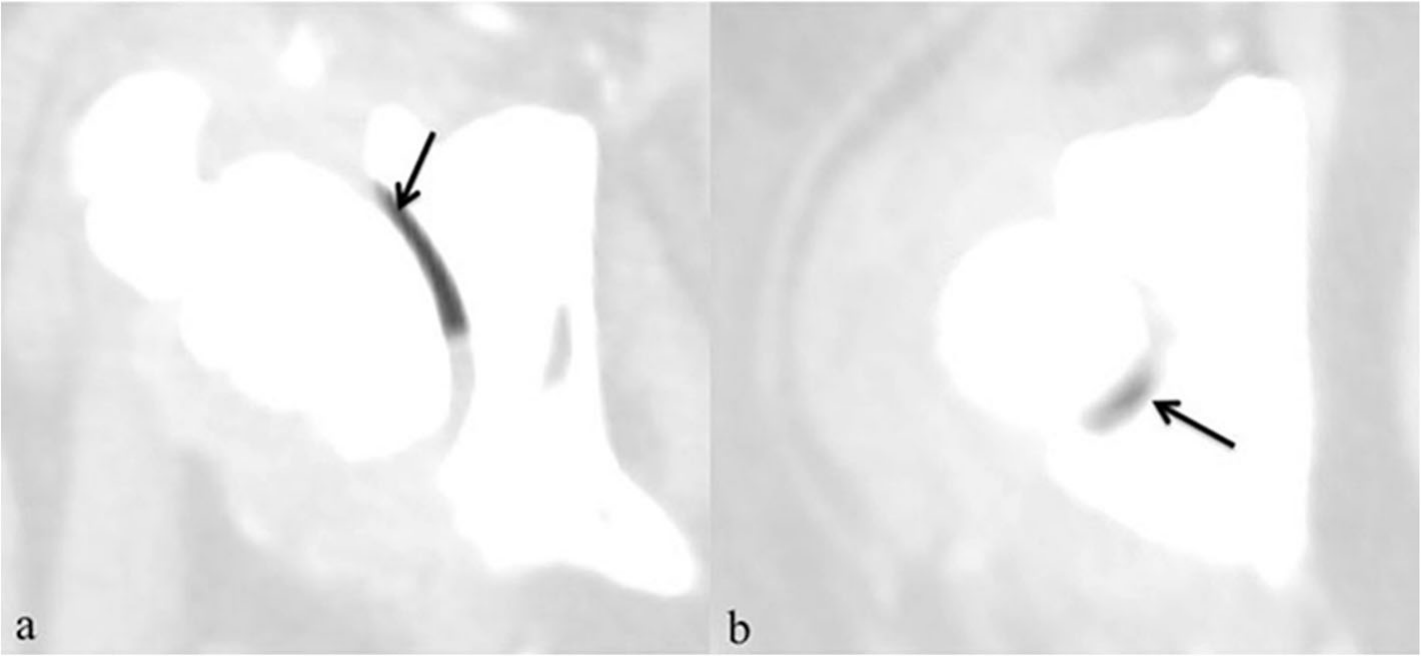
Severe degenerative coxofemoral joint and presence of gas (vacuum phenomenon; arrows) in coxofemoral joint in axial CT images (pulmonary window); a: Dog in prone position; b: Dog in lateral position

VP was detected at the laterodorsal area of the affected acetabulum in the prone position for 24 dogs (Fig. 1a) and in the lateral recumbent position for one dog (Fig. 1b). Comparing the breeds of the canine patients, large breed dogs were more affected by VP (20 of 25 dogs had VP [80.00%]) than medium (3 of 25 dogs had VP [12.00%]) and small (2 of 25 dogs had VP [8.00%]) breed dogs. By G2 DJD joints, VP were more likely to be detected in large breed dogs (23/64 joints [35.94%]), followed by small (6/35 joints [17.14%]) and medium (2/24 joints [8.33%]) breed dogs.

A significant association was found between the total presence of DJD and coxofemoral VP in large breed dogs (OR = 17.58, CI 2.32–133.42), whereas a similar association was not found for small and medium breed dogs (OR = 1.50, CI 0.15–12.64 and OR = 0.95, CI 0.09–9.54, respectively).

## Discussion

The results of this study showed the prevalence, characteristics and relationship between VP and coxofemoral DJD in dogs by using CT. Although VP is a common finding in the human coxofemoral joint, its description in dogs with coxofemoral degenerative disease is uncommon. Several imaging modalities, including radiography, CT, and MRI, can be used to detect VP lesions [16]. However, the gold standard for VP detection is CT [16] because it is more sensitive in detecting gas bubbles in joint spaces, especially using the pulmonary window. VP is associated with DJD at several locations [13, 24, 27], and the coxofemoral joint is one of the predominant locations of DJD development in canine patients [10]. Because CT is becoming increasingly available in veterinary practices, a higher incidence of VP may be found in animals. As coxofemoral VP has been detected only in human FAI [21] or DDH [17], the detection of VP in the canine coxofemoral joint could provide further information for characterizing the severity of CHD in dogs. With the distracted hip position [5], VP might be one of the additional factors used to detect DJD at the early stage when coxofemoral subchondral bone changes are not obviously detected on radiograph. However, the utilization of VP as the DJD prediction, further studies must be investigated. To our knowledge, there have been no previous reports of VP in dogs with coxofemoral joint DJD.

Most of the population in this study comprised small breed companion dogs owned by individuals residing in urban areas. However, a higher prevalence of DJD was found in large breeds than in small and medium breeds. One of the most common causes of DJD in large or giant breed dogs is CHD. Large and giant breed dogs are more predisposed to CHD than smaller breed dogs. It has been reported that 74% of the large breed dog population is affected by CHD [28]. CHD is characterized by abnormal development of the hip joint, which leads to joint laxity and may progress to DJD [2–5, 29]. The cause of CHD remains unclear; however, some reports have described genetic malformation(s) and other multifactorial sources [7, 30]. In humans, the disease is commonly found in juvenile patients and is known as DDH; it is highly prevalent (84.1%) in girls [31]. In contrast, CHD has no sex predominance in dogs [32]. However, the clinical signs of CHD vary and are different in each dog, ranging from lameness and pain to voluntary paresis [29], which negatively impacts quality of life.

All cases of VP in this study were detected in dogs with established coxofemoral joint DJD. Furthermore, VP in conjunction with coxofemoral DJD was found to be particularly prevalent among large breed dogs. These results suggest a more significant association between DJD and VP in large breed dogs than in small and medium breed dogs. In a previous study, VP was found mainly in middle-aged to geriatric canine patients affected by intervertebral disc disease [33]. However, this study did not find a correlation between age and the presence of VP. This may be due to the predisposition of large and giant breed dogs to CHD, which can develop early in life; for example, high-grade DJD could presumably develop in young dogs as well.

Regarding the coxofemoral joint being affected by DJD, it may be assumed that intra-articular gas (mainly nitrogen, oxygen, carbon, and other gases) from the surrounding tissue is unable to dissolve in the joint space. This can be explained by the combination of anatomy and physics [18]. Based on Henry and Boyle’s Law, an increase in the joint space leads to volume expansion and subsequently induces negative pressure, which decreases the solubility of gas from the surrounding tissue [34]. Accordingly, gas leaves precipitate out of intra-articular solution or synovial fluid [16]. Therefore, VP can be elucidated by any process that allows increased articular volume, such as applying excessive distraction on joints. VP can occur if the traction force is between 400 and 600 N, such as during hip arthroscopy [21]. In dogs, using the PennHIP method during radiography [25] or coxofemoral joint laxity could lead to VP [5]. However, VP gas can be reabsorbed into the nearest tissues when the joint has returned to a new equilibrium of volume and pressure after the compression or traction force is reduced [27].

Because of the retrospective nature of this study, the positioning of the dogs during CT examination could not be controlled. Previous studies involving human subjects have reported that VP of the coxofemoral joint was only visible through plain radiography when the patients were in the “frog-leg” or supine position. These positions can increase the volume of the hip joint cavity and enable VP detection [17]. Coxofemoral VP was only found in 5.49% of all enrolled dogs in the current study, and 24/25 (96%) dogs affected by VP were examined in the prone position (only 1/25 in the lateral position). Although VP was detected only in the advanced stage of DJD in this study, the use of proper positions for stress and traction such as the frog-leg position for CT combined with MPR to better visualize the relationship between the femoral head and the acetabulum may result in a higher detection rate of VP in the canine coxofemoral joint, especially in dogs with a mild degree of DJD.

The relevance of degenerative signs for VP remains unclear. VP has been reported in dogs to indicate vertebral disc degeneration and herniation; however, this is sufficiently accurate [8, 33, 35]. Moreover, there are no relevant reports describing canine coxofemoral DJD and VP signs. Due to the retrospective nature of the present study, we could not evaluate the correlation between the clinical signs of hindlimb and VP at the coxofemoral joint. Further studies investigating all clinical signs and radiographic examinations performed in different positions compared with those observed using CT scans including studies investigating breed-specific groups, would provide more information between VP and the degree of DJD and/or CHD.

## Conclusions

Coxofemoral VP is more prone to be detected on CT in dogs with G2 DJD or established DJD. Moreover, it was predominantly observed in large breed dogs with no association with patient age. Although coxofemoral VP is detected on CT imaging using a pulmonary window, specific patient positioning could enhance the number of occurrences and size of VP in the coxofemoral joint, enabling better detection. Further prospective studies are needed to obtain more information for clinical diagnosis, especially with regard to CHD.

## Methods

### Animals

CT reports of canine patients submitted to the Diagnostic Imaging Unit, The Small Animal Teaching Hospital, Faculty of Veterinary Science, Chulalongkorn University (Bangkok, Thailand) between May 2013 and April 2018, were retrospectively examined. All CT images, regardless of animal positioning, for mature canine patients who required CT scanning for orthopedic or nonorthopedic purposes were included. Examples of nonorthopedic purposes included whole abdominal and perineum examinations and whole-body metastatic screening. Dogs were excluded if CT examination reported pelvic fracture, infection or tumors and/or if the dogs had undergone recent pelvic or hindlimb surgery, if the dogs had received intra-articular injections of the coxofemoral joint, or if the imaging did not cover the entire pelvic region. All clinical data, including breed, age, sex (including gonadal status), and body weight of all included dogs, were recorded.

### Analysis of CT images

**Fig. 2:**
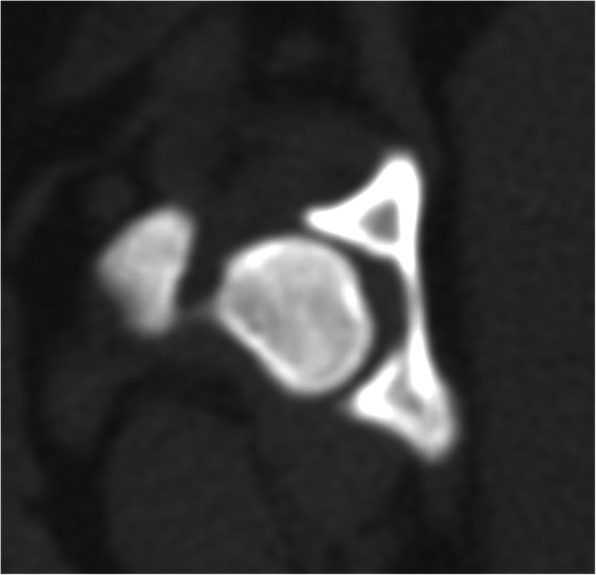
No coxofemoral joint DJD lesion (Grade 0 or G0) in axial CT images (bone window): Femoral head and acetabulum are congruent and no lesion of DJD

**Fig. 3:**
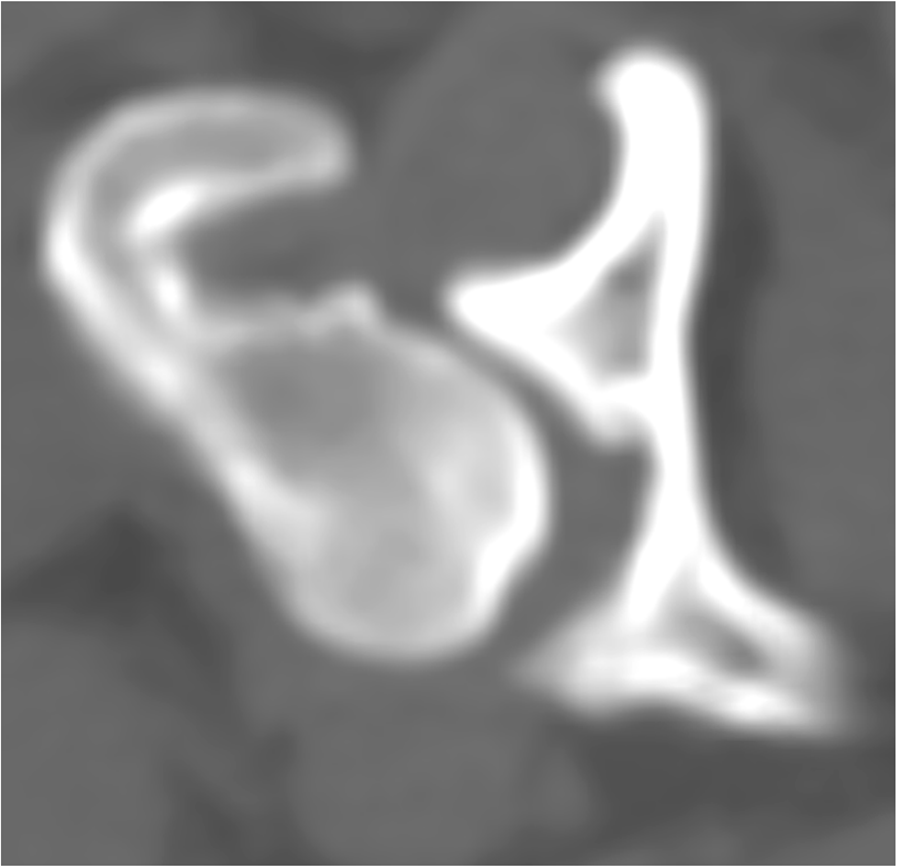
Developing coxofemoral joint DJD lesion (Grade 1 or G1) in axial CT images (bone window): Osteophyte formation at dorsal femoral head

**Fig. 4:**
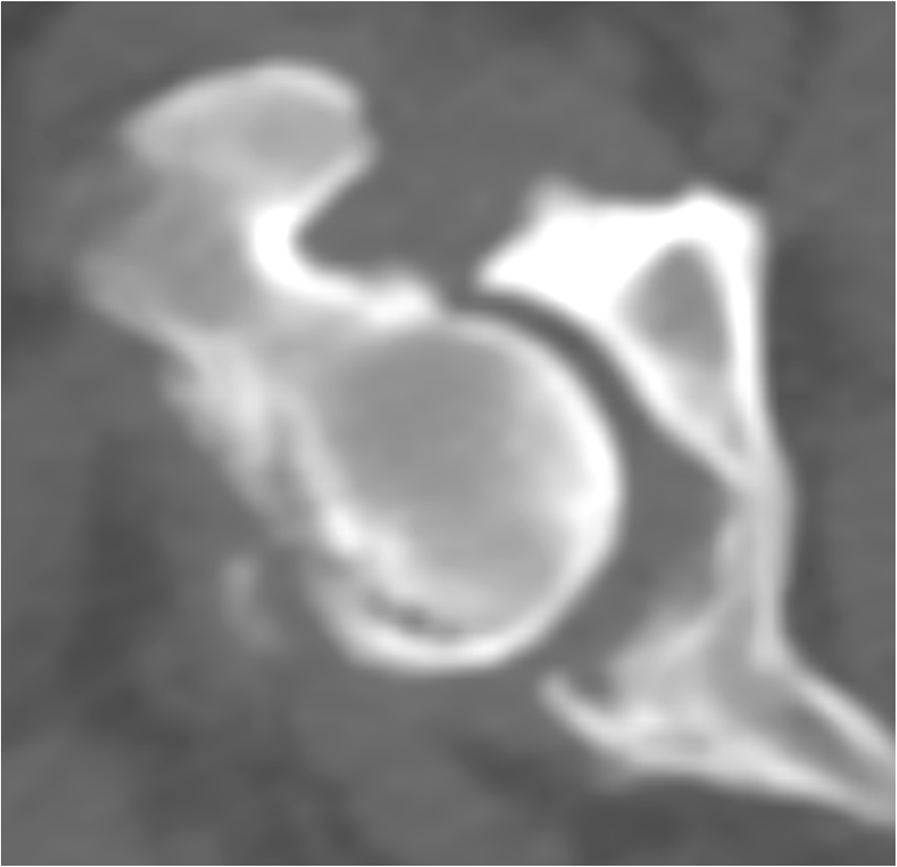
Established coxofemoral joint DJD lesion (Grade 2 or G2) in axial CT images (bone window): Severe sclerosis of head and acetabulum with femoral head and acetabulum incongruency

All CT images were retrieved from the hospital radiological system in Digital and Communications in Medicine (DICOM) format and reanalyzed using DICOM viewer software (Osirix, Geneva, Switzerland) at a non-CT unit work station with a 2560 × 1440 monitor. All CT images that included the pelvic region were acquired using a 64-slice, helical CT scanner (Optima CT660, GE Healthcare, Japan) in the prone, supine, or lateral positions. CT images were contiguous and transverse, and the field of view was dependent on the body size of the dog. The matrix size of CT imaging was 512 × 512. The CT slice thickness ranged from 0.625 to 2.5 mm and was dependent on the size of the dog, which did not interfere with the interpretation process. The CT images of each dog were analyzed to categorize DJD changes at the coxofemoral joint using a bone window (window width (WW), 1500 Hounsfield units (HU); window level (WL), 300 HU). The selected dogs were classified into three grades [36]: G0: no coxofemoral DJD lesion, as the femoral head and acetabulum are congruent (Fig. 2); G1: developing coxofemoral DJD lesion, only presenting osteophyte at the dorsal acetabular rim or femoral head (Fig. 3); and G2: established coxofemoral DJD lesion, presenting severe sclerosis and incongruency of the femoral head and acetabulum (Fig. 4).

Subsequently, evidence of intracoxofemoral gas was detected using a pulmonary window at 1400 HU WW and − 500 HUWL. This process included multiplanar reconstruction (MPR) application. Dogs with a hypoattenuated area at approximately ≤ -300 HU [20] were considered to be affected by intra-articular gas (i.e., VP).

### Statistical analysis

Statistical analyses were performed using GraphPad statistical software (GraphPad Software, CA, USA). All clinical data were submitted to descriptive analysis. Before performing a statistical comparison of each parameter, the normality test for each data set was performed by the Shapiro Wilk test. The presence of DJD of the hip in each breed is presented as a percentage. The relationships between age and DJD and between age and VP were assessed using the Spearman correlation test. The relationship and association between the presence of DJD and VP was assessed using the odds ratio (OR). Significance was set at *P ≤ 0.05* for all tests.

## Data Availability

The datasets used and/or analysed during the current study are available from the corresponding author on reasonable request.

## Abbreviations

DJD: Degenerative joint disease
CHD: Canine hip dysplasia
CT: Computed tomography
DDH: Developmental dysplasia of the hip
FAI: Femoroacetabular impingement
HU: Hounsfield unit
MPR: Multiplanar reconstruction
MRI: Magnetic resonance imaging
US: Ultrasonography
VP: Vacuum phenomenon
WL: Window level
WW: Window width
N: Newton
AKC: American Kennel Club
